# Assessment of the Robustness of Convolutional Neural Networks in Labeling Noise by Using Chest X-Ray Images From Multiple Centers

**DOI:** 10.2196/18089

**Published:** 2020-08-04

**Authors:** Ryoungwoo Jang, Namkug Kim, Miso Jang, Kyung Hwa Lee, Sang Min Lee, Kyung Hee Lee, Han Na Noh, Joon Beom Seo

**Affiliations:** 1 Department of Biomedical Engineering College of Medicine University of Ulsan Seoul Republic of Korea; 2 Department of Convergence Medicine Asan Medical Center University of Ulsan College of Medicine Seoul Republic of Korea; 3 Department of Radiology Asan Medical Center University of Ulsan College of Medicine Seoul Republic of Korea; 4 Department of Radiology Seoul National University Bundang Hospital Seoul National University College of Medicine Seongnam Republic of Korea; 5 Department of Health Screening and Promotion Center Asan Medical Center Seoul Republic of Korea

**Keywords:** deep learning, convolutional neural network, NIH dataset, CheXpert dataset, robustness

## Abstract

**Background:**

Computer-aided diagnosis on chest x-ray images using deep learning is a widely studied modality in medicine. Many studies are based on public datasets, such as the National Institutes of Health (NIH) dataset and the Stanford CheXpert dataset. However, these datasets are preprocessed by classical natural language processing, which may cause a certain extent of label errors.

**Objective:**

This study aimed to investigate the robustness of deep convolutional neural networks (CNNs) for binary classification of posteroanterior chest x-ray through random incorrect labeling.

**Methods:**

We trained and validated the CNN architecture with different noise levels of labels in 3 datasets, namely, Asan Medical Center-Seoul National University Bundang Hospital (AMC-SNUBH), NIH, and CheXpert, and tested the models with each test set. Diseases of each chest x-ray in our dataset were confirmed by a thoracic radiologist using computed tomography (CT). Receiver operating characteristic (ROC) and area under the curve (AUC) were evaluated in each test. Randomly chosen chest x-rays of public datasets were evaluated by 3 physicians and 1 thoracic radiologist.

**Results:**

In comparison with the public datasets of NIH and CheXpert, where AUCs did not significantly drop to 16%, the AUC of the AMC-SNUBH dataset significantly decreased from 2% label noise. Evaluation of the public datasets by 3 physicians and 1 thoracic radiologist showed an accuracy of 65%-80%.

**Conclusions:**

The deep learning–based computer-aided diagnosis model is sensitive to label noise, and computer-aided diagnosis with inaccurate labels is not credible. Furthermore, open datasets such as NIH and CheXpert need to be distilled before being used for deep learning–based computer-aided diagnosis.

## Introduction

Posteroanterior chest x-ray (CXR) is one of the most widely used methods to evaluate a subject’s chest. CXR is low cost and easy to assess and acquire, and it provides a variety of information. Researchers developed computer-aided diagnosis (CAD) algorithms for CXRs because of the substantial presence of CXRs in large hospitals and medical centers [1]. At present, there are no widely used clinically meaningful CAD algorithms with classical image processing algorithms. However, the success of deep learning has led to the development of deep learning–based CXR CAD algorithms [2]. Among the various types of deep learning algorithms, the convolutional neural network (CNN) is the most widely used technique for CXR classification.

Before applying CNN to CAD development, we need to consider the robustness of CNN for inaccurate datasets. It is believed that CNN is robust to label noise [3]. Conversely, clean labels and accurate datasets are considered necessary conditions for CNN-based classification. However, the differences in complexity between datasets from Modified National Institute of Standards and Technology (MNIST) and CXRs were enormous. The MNIST images had a size of 28×28 pixels, whereas the image sizes in CXR datasets were mostly above 1024×1024 pixels. Therefore, relying on the robustness of deep learning alone for CXR datasets would be insufficient. Some [3] asserted that accuracy over 90% with 0% noisy labels is not very different from an approximate accuracy of 85% with 90% noisy labels. However, in medicine, an accurate diagnosis is essential for appropriate treatment, and even a 1% decrease in accuracy cannot be tolerated.

Since open CXR datasets from the National Institutes of Health (NIH) and Stanford CheXpert are preprocessed using natural language processing, they tend to contain [4] a certain extent of wrong and uncertain labels [5,6]. Several groups studied the effect of label noise in the CNN classification model. Rolnick et al [3] claimed that CNNs are robust to massive label noise. Beigman and Beigman [4], Guan et al [7], Lee et al [8], Choi et al [9], and Sukhbaatar and Fergus [10] attempted to develop models from noisy datasets directly. Others such as Brodley and Friedl [11] identified and reduced noisy data using majority voting before training. This research claims that they can make a model robust for up to 30% of label noise. This type of research is subject to the risk of classifying hard labels as noisy labels. To overcome this problem, some researchers attempted to combine noisy data with accurate datasets, as proposed by Zhu [12]. When the label noise was provided, Bootkrajang and Kabán [13] proposed a generic unbiased estimator for binary classification. Unlike electronic health records, images can be re-reported any time with domain experts’ efforts. There are several studies that analyzed electronic health records using natural language processing techniques [14,15].

Many have attempted to classify CXR with deep learning techniques. Rajpurkar et al [5] proposed a CNN-based CXR classifier with an overall area under the curve (AUC) ranging between 0.8 and 0.93. Yao et al [16] used a similar method to classify multiclass CXR. Pesce et al [17] used over 430,000 CXRs and proposed an architecture with attention structure based on the evidence that deep learning is robust to label noise [3].

The questions raised were “Are noisy and wrong-labeled datasets credible?” and “Can we believe a CAD model that used these open datasets during training?” In this study, we contemplate the credibility of these datasets and the effect of label noise during training. The aim of this study is threefold: (1) to train computed tomography (CT)-confirmed CXR datasets from Asan Medical Center (AMC) and Seoul National University Bundang Hospital (SNUBH), which can be considered clean with an intentionally given label noise of 0%, 1%, 2%, 4%, 8%, 16%, and 32%; (2) to train NIH and CheXpert datasets, which are considered noisy with an intentionally given label noise of 0%, 1%, 2%, 4%, 8%, 16%, and 32%; and (3) to have the NIH and CheXpert datasets re-evaluated by 3 physicians and one radiologist.

## Methods

### Image Dataset

Our CXRs were collected from 2 hospitals, AMC and SNUBH in South Korea. Data from 2011 to 2016 were collected. Every CXR was confirmed with its nearest corresponding CT scan and was reevaluated by a chest radiologist with more than 20 years of experience. CXRs contained 5 clinically relevant disease categories, namely, nodule (ND), consolidation (CS), interstitial opacity (IO), pleural effusion (PLE), and pneumothorax (PT). These categories were classified into 2 classes, normal and abnormal. A detailed description of our dataset is provided in Multimedia Appendix 1.

Descriptions of the NIH and the CheXpert datasets can be found in Multimedia Appendices 2 and 3 [6,18]. To validate the NIH and CheXpert datasets, we randomly sampled the same number of normal and abnormal images from the NIH and CheXpert datasets as that from our dataset, that is, all 3 datasets were sampled to have 7103 no finding images and 8680 abnormal images. In the NIH dataset, images were classified into 15 categories including a “no finding” category. For the NIH dataset, we did not distinguish each disease category, but unified all the disease categories into 1 class, “abnormal”. In the CheXpert dataset, images were classified into 14 categories including “no finding.” In each image class, every image was subclassified as positive/uncertain/negative. We did not use positive/uncertain/negative because the uncertain class can be confusing and negative images were not clinically important. Instead, 14 positive-labeled disease categories were classified as “abnormal,” and the “no finding” category was classified as “normal” in the CheXpert dataset. Because there were disease categories present in the CheXpert dataset, which were not in our dataset or the NIH dataset, we unified every disease class as “abnormal” and considered “no finding” as “normal.” Furthermore, the “abnormal” class was randomly sampled to be the same number as our “abnormal” dataset without considering the number of each disease class. These “no finding” and “abnormal” dataset descriptions are presented in Table 1.

**Table 1:** Brief description of the datasets of Asan Medical Center and Seoul National University Bundang Hospital, National Institutes of Health, and CheXpert.

Distribution of images	AMC^a^ and SNUBH^b^ dataset	NIH^c^ dataset	CheXpert dataset
Number of no-finding or normal images	7103	60,361	22,419
Number of abnormal images	8680	51,759	201,897
Number of total images	15,783	112,120	224,316

^a^AMC: Asan Medical Center.

^b^SNUBH: Seoul National University Bundang Hospital.

^c^NIH: National Institutes of Health.

After random shuffling, we analyzed the distribution of 3 randomly shuffled datasets. The distributions of these randomly shuffled datasets are shown in Multimedia Appendix 4.

The label quality of public data from open datasets was evaluated by 3 licensed nonradiologist physicians and 1 board-certified radiologist. For the 3 nonradiologists, in each of the CheXpert and the NIH dataset, we randomly sampled 100 images. In the NIH dataset, 25 images were “abnormal” and 75 images were “no finding.” In CheXpert, 85 images were “abnormal” and 15 images were “normal.” For the radiologist, we randomly selected 200 images from each public dataset. The board-certified radiologist evaluated each given dataset twice, and we recorded the concordance rate for the 2 evaluations. For each open dataset, these images were passed to 3 physicians and 1 radiologist, who reported whether each image belonged to the “no finding” or “abnormal” category.

### Image Preprocessing

Every CXR image from the NIH and CheXpert datasets was stored in an 8-bit PNG format. To feed the images in the training model, we changed 3- or 4-channel PNG images to grayscale. The 12-bit DICOM (Digital Imaging and Communications in Medicine) files in our dataset were converted into 8-bit gray PNG format, for which we attempted to set a consistent training condition. In open datasets, sizes of images differed from image to image. To solve this problem, we unified the image size to be 1024×1024 pixels. Similarly, our DICOM images were resized from approximately 2000×2000 pixels to 1024×1024 pixels. Bilinear interpolation was used to resize images, and min-max scaling was applied to each image so that every pixel had a value in the range of 0-1. All the processing was performed using the opencv-python package by Olli-Pekka Heinisuo.

### Training Details

Each dataset was classified into 3 groups: training, validation, and test sets. The detailed composition of our dataset including the training, validation, and test sets is presented in Multimedia Appendix 5. Among the various CNN models, CheXNet by Rajpurkar et al [5] was selected as the baseline model. CheXNet is a 121-layered Densenet [19] with 14 disease categories. We changed the last fully connected layer to 1 node to simplify the classification into normal and abnormal. We trained CheXNet from scratch without using the pretrained model. Labels of each training dataset were intentionally misrepresented with rates of 0%, 1%, 2%, 4%, 8%, 16%, and 32%. To generate a training set to have every label noise, we first randomly shuffled all the datasets and changed the label of images in the shuffled list in order from the front. The order was shuffled again to distribute the misrepresented label data evenly in the entire training set. We used Keras python package and Adam optimizer [20] with a learning rate of 0.0001. The loss was set to be binary cross-entropy, and we measured the accuracy with a threshold of 0.5. We trained 20 epochs for each label noise level and each dataset. The training was conducted with a NVIDIA GeForce RTX 2070 for approximately 3 days for each dataset. Moreover, we did not apply label noises for the validation and test sets.

### Evaluation Metric and Statistics

For inference, we selected the model with the smallest validation loss in each dataset. In each test set of datasets, we evaluated receiver operating characteristics (ROC) and AUC. The inference results were compared using a semi-log plot. Subsequently, AUC of 0% was compared with each noise level, using standard error defined by Hanley and McNeil [21]. The SE is defined as follows:


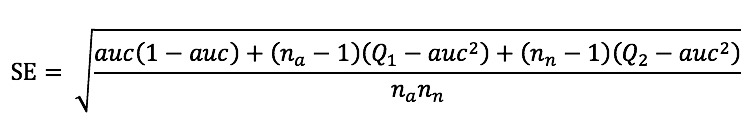


where auc is AUC, n_a_ is the number of abnormal images, and n_n_ is the number of normal images, 
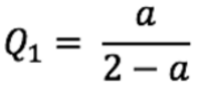
 and 
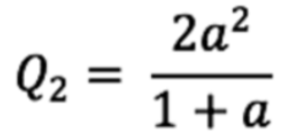


## Results

### Accuracies of Each Label Noise

**Figure 1:**
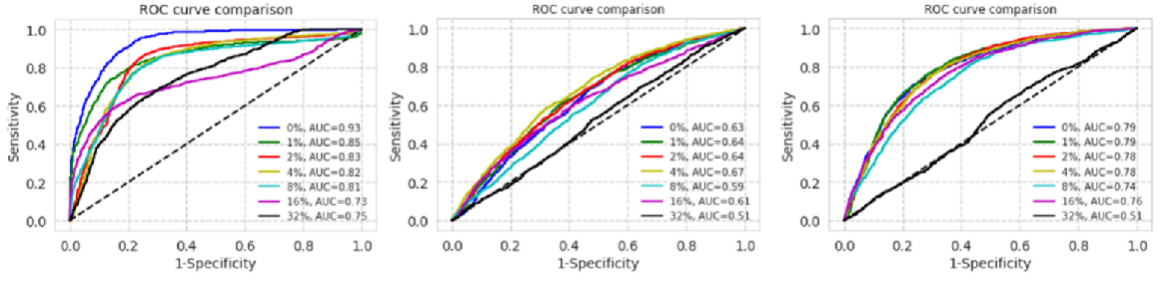
Receiver operating characteristic (ROC) curves for datasets of Asan Medical Center and Seoul National University Bundang Hospital, National Institutes of Health, and CheXpert (from left to right) with each label noise rate (0%, 1%, 2%, 4%, 8%, 16%, and 32%).

After training 3 datasets with the CNN architecture, ROC curves were drawn as depicted in Figure 1.

**Figure 2:**
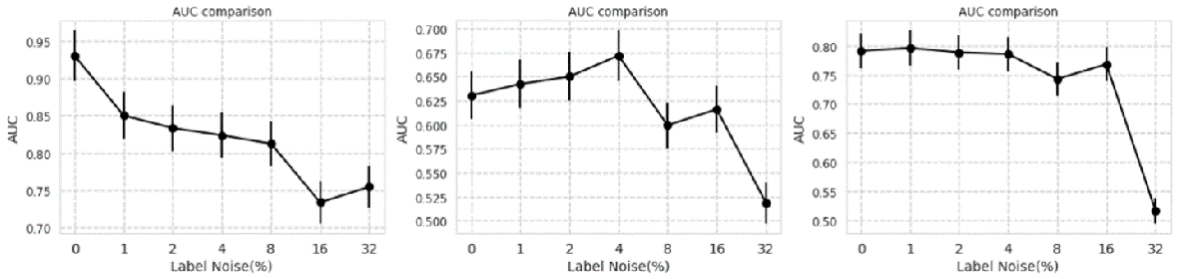
Semilog plot of area under the curves (AUC) of receiver operating characteristic (ROC) curves in the datasets of Asan Medical Center and Seoul National University Bundang Hospital, National Institutes of Health, and CheXpert (from left to right) with each label noise rate (0%, 1%, 2%, 4%, 8%, 16%, and 32%).

Figure 2 illustrates a semilog plot of AUCs of ROC curves from our dataset, the NIH dataset, and the CheXpert dataset for every noise level. Each vertical line means standard error for given AUC.

**Figure 3:**
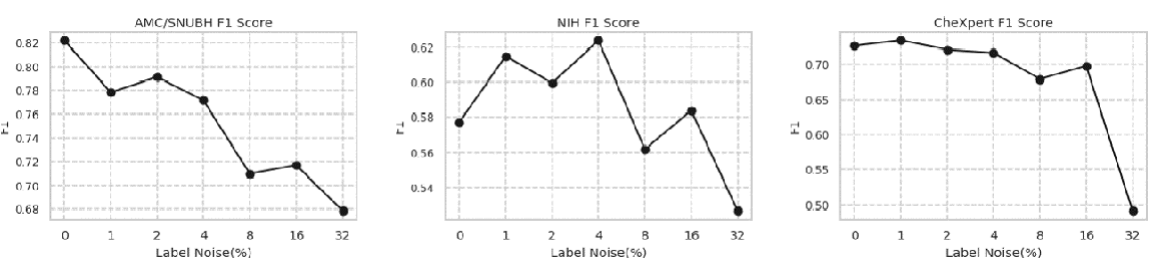
F1 scores of the datasets of Asan Medical Center and Seoul National University Bundang Hospital, National Institutes of Health, and CheXpert (from left to right).

In the NIH and the CheXpert datasets AUC was poorer than that in our dataset at 0% label noise. The AUC of our dataset was more sensitive to label noise than that of the NIH and the CheXpert datasets. F1 scores are plotted in Figure 3.

The ROC comparisons for the 3 datasets are presented in Table 2. It became statistically significant when noise level became 2% in our dataset. However, in the NIH and CheXpert datasets, there was no statistical significance until 16% of noise was observable in the training set.

**Table 2:** Receiver operating characteristic (ROC) comparison for the datasets of Asan Medical Center and Seoul National University Bundang Hospital, National Institutes of Health, and CheXpert.

Dataset and label noise level (%)		Difference of AUC^a^ with respect to 0%	*P* value
**AMC^b^** **and SNUBH^c^**			
	1	0.08	.08
	2	0.097	.04
	4	0.107	.02
	8	0.118	.007
	16	0.197	<.001
	32	0.176	<.001
**NIH^d^**			
	1	–0.012	.74
	2	–0.020	.58
	4	–0.041	.24
	8	0.031	.37
	16	0.014	.68
	32	0.111	<.001
**CheXpert**			
	1	–0.005	.91
	2	0.003	.99
	4	0.005	.90
	8	0.048	.86
	16	0.022	.94
	32	0.028	<.001

^a^AUC: area under the curve.

^b^AMC: Asan Medical Center.

^c^SNUBH: Seoul National University Bundang Hospital.

^d^NIH: National Institutes of Health.

**Figure 4:**
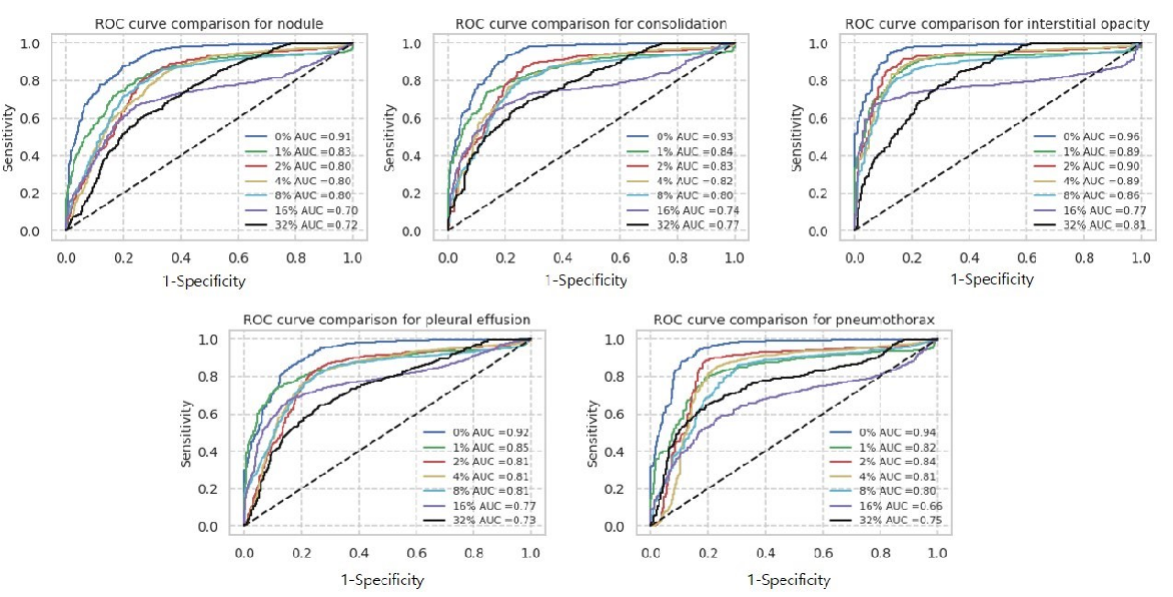
Subgroup analysis of abnormal cases in the dataset of Asan Medical Center and Seoul National University Bundang Hospital.

For our dataset, we analyzed subgroups of abnormal cases. It is shown in Figure 4.

There were 1413 normal CXRs, 449 ND CXRs, 322 CS CXRs, 261 IO CXRs, 548 PLE CXRs, 298 PT CXRs in our test set. We joined 1413 normal data with each disease subclass and performed ROC curve analysis. For overall subgroups including ND, CS, IO, PLE, PT, there was no distinguishing subgroup, which was much more sensitive to label noise. However, among these classes, IO was most robust to label noise, showing low decline of AUCs.

### Visual Scoring of Open Dataset

**Figure 5:**
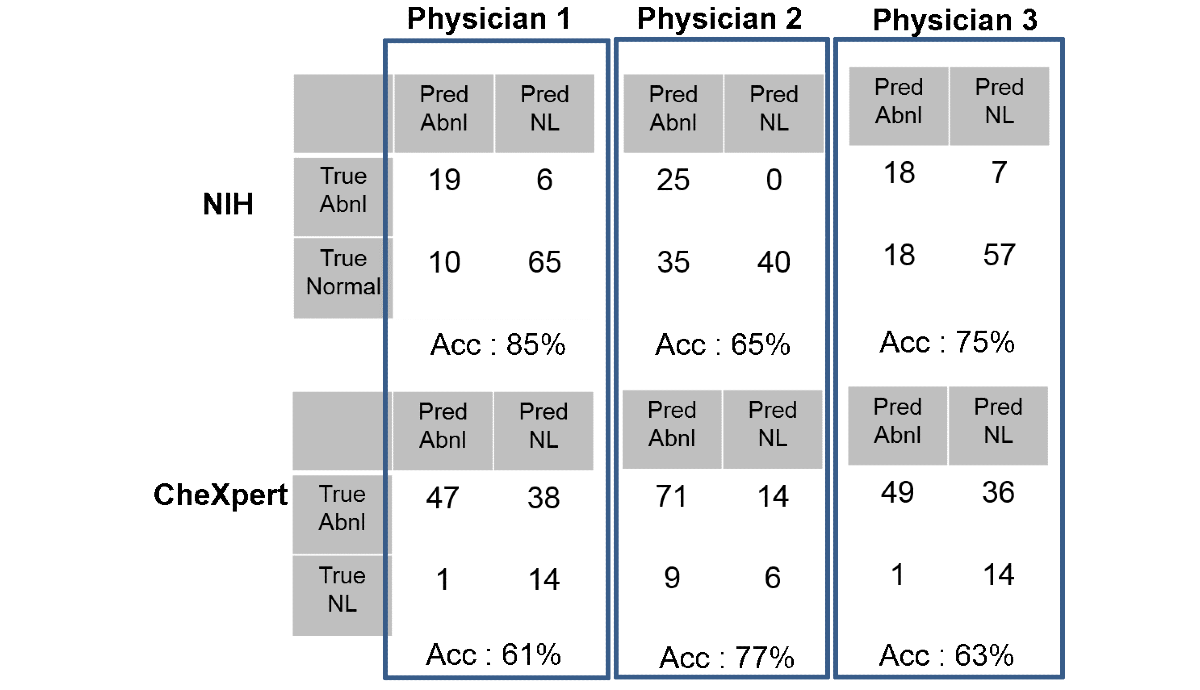
Visual scoring by 3 licensed physicians. Pred: predicted; Abnl: abnormal; NL: normal; NIH: National Institutes of Health; Acc: accuracy.

The NIH and the CheXpert datasets were reevaluated by 3 nonradiologist licensed physicians and 1 radiologist. The physicians evaluated CXRs once for each doctor, and the radiologist evaluated CXRs twice. The 3 physicians rated the accuracy of the NIH dataset as 75% (75/100), 65% (65/100), and, 84% (84/100), and that of the CheXpert dataset as 65% (65/100), 77% (77/100) and 61% (61/100), respectively. The radiologist who evaluated CXRs twice rated the accuracy of NIH dataset as 67.5% (135/200) and 65 % (130/200) for each evaluation and rated the accuracy of CheXpert dataset as 81% (162/200) and 77% (154/200) for each evaluation. The concordance rates of 2 evaluations for 2 datasets were 92% (184/200) and 56% (112/200) for the NIH and CheXpert datasets, respectively. Figure 5 depicts the sensitivity and specificity of the report of the 3 physicians. First row is the result of visual scoring by 3 physicians for the NIH dataset, and the second row is the result of visual scoring by 3 physicians for the CheXpert (Stanford) dataset.

**Figure 6:**
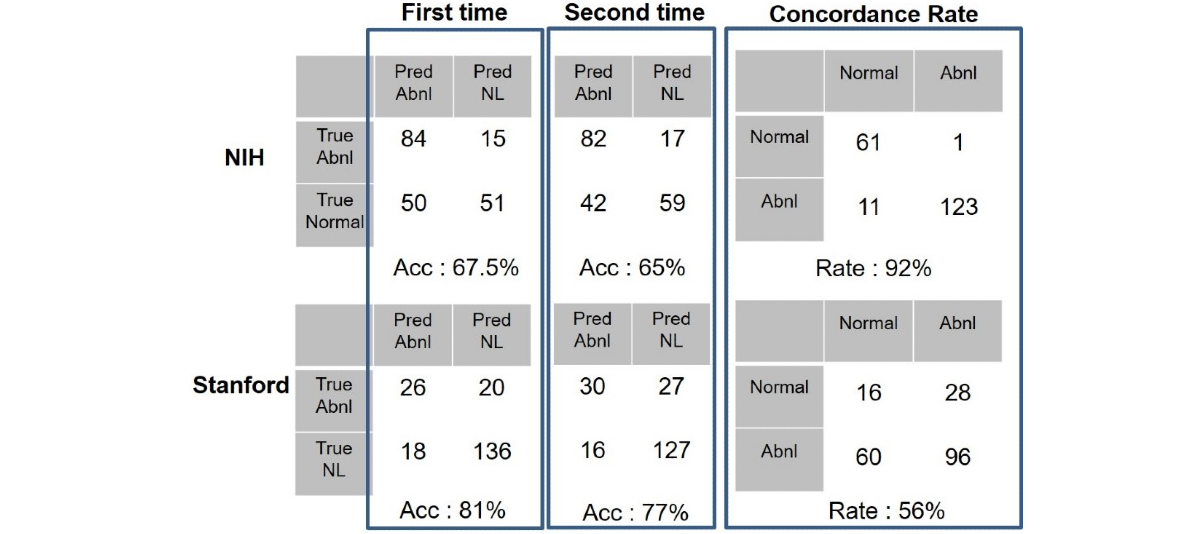
Visual scoring of thoracic radiologist over a 20-year experience. Pred: predicted; Abnl: abnormal; NL: normal; NIH: National Institutes of Health; Acc: accuracy.

Figure 6 shows the accuracy, sensitivity, specificity of 2 evaluations of 1 radiologist with the concordance rate of 2 evaluations. One radiologist had visually scored 2 public datasets twice. First and second columns from the left show the result of visual scoring for the public datasets. The third column is about concordance rate for the 2 visual scorings for each dataset.

## Discussion

The results of our dataset reveal that the CNN architecture is extremely sensitive to label noise. However, the results of the NIH and CheXpert datasets demonstrate that open datasets are robust to label noise, suggesting that the NIH and CheXpert datasets essentially contain label noises. These datasets do not significantly change the label noise levels and yield robustness despite the label noise. Therefore, training open datasets with CNN architectures has several drawbacks. First, CheXNet cannot be trained in the NIH dataset, because of extensive noise level of NIH dataset. Since open datasets were processed with classical natural language processing, abnormal CXRs were reported to have “no interval change” can be categorized as “no findings.” This can amplify label noise of open datasets.

Furthermore, the “no finding” category does not imply normal. There were 15 classes in NIH classified as “no finding,” and 14 classes in CheXpert classified as “no finding,” suggesting that other lesions may be categorized as “no finding.” For example, cavity due to tuberculosis, reticular pattern due to diffuse interstitial lung diseases, hyperinflation due to chronic obstructive lung diseases could be classified as “no finding.” Rajpurkar et al [5] reported the CheXNet performance to be similar to that of a radiologist in categorizing pneumonia, rather than a “no finding” category, possibly caused by label noises and/or due to the insufficient performance of CheXNet for differentiating “no finding” and “abnormal.” Therefore, labeling with natural language processing is not suitable for CXR CAD model development. Rating accuracies of our 3 physicians on “no finding” and “abnormal” was approximately 60%-80%, and the accuracy of confirmation by 1 radiologist for the NIH and CheXpert dataset was around 60% and 80%, respectively, which implies that these open datasets have a high occurrence of mislabeled data. The concordance rate of 1 radiologist was 92% (184/200) for NIH and 56% (112/200) for CheXpert. This low concordance rate for CheXpert may have originated from blurry texture of CheXpert images.

To analyze their performance, we experimented the ability of corrected test set of open datasets. First, after the radiologist’s 2-time confirmation, we tested corrected labels using weights of model that were trained with each label noise. The result is shown in Multimedia Appendix 6. Due to the massive label noise of NIH dataset, CheXNet does not work properly for each model of label noise. In CheXpert settings, situation is little bit better yet performance was poor as expected.

There could be an array of additional issues that affect the quality of the open datasets. The CheXpert and NIH datasets are 8-bit PNG image files. Therefore, information loss is unavoidable during conversion from 12-bit DICOM files to the PNG image format.

Robustness of the CheXNet model trained by the NIH and CheXpert datasets does not translate to the robustness of the CNN architecture. The results of our dataset show that CNN is not robust to the noise level. Rather, robustness of the models trained by open datasets can be considered a result of their original impurity. The open datasets are not well-preprocessed, leading them to contain label errors to a certain extent. A low level of label noise does not visibly affect the impurity, and accuracy seems to endure up to 16%.

Regardless of these drawbacks, CNN is considered the best tool for CAD development. Our study urges CAD developers to maximize their effort in accumulating extremely high-quality datasets.

Our study has several limitations. First, we considered only 1 network, CheXNet. Other networks such as ChoiceNet can be robust to label noise [9]. Second, a well-performing model that is robust to label noise is not indicative of its tolerability towards label noise in open datasets. Using open datasets commercially or for research must be seriously considered. Unlike MNIST, they have considerable impacts on the diagnosis of each patient.

Furthermore, it is interesting to speculate active learning with predicted images, which have low confidence levels. That is, predicted labels that have low confidence rate after final activation function, such as 0.4 to 0.6. We might consider them as mislabeled images. Therefore, using high-confidence images and their labels, we can re-label low confidence images assisted by radiologist if needed and train CNN again. This can be used as strategy for training the noisy dataset accurately. However, this strategy is beyond the scope of this study. In our future work, this kind of strategy will be used to train noisy dataset accurately.

As mentioned earlier, even a 1% decrease in accuracy can have an enormous effect on a large patient group. Additionally, categorizing data into “no finding” and “abnormal” may not be ideal as this could be a direct consequence of mislabels on “no finding.” There may be other disease patterns that were not labeled, resulting in an unfair comparison of the 3 datasets with the same criteria. Furthermore, there is a statistical limitation for this study. To compare CNN models exactly, we trained models with only 20 epochs for each label noise level. For some training steps, 20 epochs did not seem sufficient for accuracy saturation. However, we used the same network with the same hyperparameters for these comparisons. For further study, multiple and repetitive training needs to be performed.

In conclusion, the robustness of CAD to label noise with open datasets seems to be a result of their impurity caused by natural language processing. CNN is not robust to label noise in large-sized and complicated images. Therefore, it needs to be emphasized that clean labels and accurate datasets are a necessary condition for developing clinically relevant CAD in medicine.

## Appendix

Multimedia Appendix 1 Dataset description of the Asan Medical Center and Seoul National University Bundang Hospital dataset.

Multimedia Appendix 2 Dataset description of the National Institutes of Health (NIH) dataset.

Multimedia Appendix 3 Dataset description of CheXpert dataset.

Multimedia Appendix 4 Distribution of 3 randomly shuffled datasets.

Multimedia Appendix 5 Dataset description for training, validation, and test sets of the Asan Medical Center (AMC) and Seoul National University Bundang Hospital (SNUBH) dataset.

Multimedia Appendix 6 Receiver operating characteristic (ROC) curves of corrected test datasets. Left is for the NIH dataset, right is for the CheXpert dataset. One radiologist with over a 20-year experience confirmed 200 images from each dataset twice, and models that have been trained with each label noise were used to draw ROC curves.

## Abbreviations

AMC: Asan Medical Center
AUC: area under the curve
CAD: computer-aided diagnosis
CNN: convolutional neural network
CS: consolidation
CT: computed tomography
CXR: chest x-ray
DICOM: Digital Imaging and Communications in Medicine
IO: interstitial opacity
MNIST: Modified National Institute of Standards and Technology
ND: nodule
NIH: National Institutes of Health
PLE: pleural effusion
PT: pneumothorax
ROC: receiver operating characteristic
SNUBH: Seoul National University Bundang Hospital
