# Supplementary material for: Assessment of the Robustness of Convolutional Neural Networks in Labeling Noise by Using Chest X-Ray Images From Multiple Centers
Source: JMIR Med Inform. 2020 Aug 4;8(8):e18089. doi: 10.2196/18089 (PMC7435602; doi:10.2196/18089)
Supplement: Multimedia Appendix 1 [file medinform_v8i8e18089_app1.docx]

**Multimedia Appendix 1.** Dataset description of the Asan Medical Center and Seoul National University Bundang Hospital dataset.

One subject may have multiple abnormalities in given chest x-ray.

| Diagnosis | Number of subjects | |
| --- | --- | --- |
|  | AMC^a^ | SNUBH^b^ |
| Normal | 6068 | 1035 |
| ND^c^ | 1012 | 1516 |
| CS^d^ | 653 | 1114 |
| IO^e^ | 312 | 1222 |
| PLE^f^ | 1599 | 1302 |
| PT^g^ | 421 | 1083 |
| Total subjects | 9755 | 6028 |

^a^AMC: Asan Medical Center.

^b^SNUBH: Seoul National University Bundang Hospital.

^c^ND: nodule.

^d^CS: consolidation.

^e^IO: interstitial opacity:

^f^PLE: pleural effusion.

^g^PT: pneumothorax.
