# Supplementary material for: Assessment of the Robustness of Convolutional Neural Networks in Labeling Noise by Using Chest X-Ray Images From Multiple Centers
Source: JMIR Med Inform. 2020 Aug 4;8(8):e18089. doi: 10.2196/18089 (PMC7435602; doi:10.2196/18089)
Supplement: Multimedia Appendix 2 [file medinform_v8i8e18089_app2.docx]

**Multimedia Appendix 2.** Dataset description of the National Institutes of Health (NIH) dataset.

One subject may have multiple abnormalities in given chest x-ray.

| Pathology | Number of images |
| --- | --- |
| Cardiomegaly | 2776 |
| Emphysema | 2516 |
| Effusion | 13,317 |
| Nodule | 6331 |
| Hernia | 227 |
| Infiltration | 19,894 |
| Mass | 5782 |
| Consolidation | 4667 |
| Pleural thickening | 3385 |
| Pneumonia | 1431 |
| Pneumothorax | 5302 |
| Atelectasis | 11,559 |
| Fibrosis | 1686 |
| Edema | 2303 |
| No finding | 60,361 |
