# Supplementary material for: Assessment of the Robustness of Convolutional Neural Networks in Labeling Noise by Using Chest X-Ray Images From Multiple Centers
Source: JMIR Med Inform. 2020 Aug 4;8(8):e18089. doi: 10.2196/18089 (PMC7435602; doi:10.2196/18089)
Supplement: Multimedia Appendix 3 [file medinform_v8i8e18089_app3.docx]

**Multimedia Appendix 3.** Dataset description of CheXpert dataset.

One subject may have multiple abnormalities in given chest x-ray.

| Pathology | Positive | Uncertain | Negative |
| --- | --- | --- | --- |
| No finding | 16,627 | 0 | 171,014 |
| Enlarged cardomediastinum | 9020 | 10,148 | 168,473 |
| Cardiomegaly | 23,002 | 6597 | 158,042 |
| Lung lesion | 6856 | 1071 | 179,714 |
| Lung opacity | 92,669 | 4341 | 90,631 |
| Edema | 48,905 | 11,571 | 127,165 |
| Consolidation | 12,730 | 23,976 | 150,935 |
| Pneumonia | 4576 | 15,658 | 167,407 |
| Atelectasis | 29,333 | 29,377 | 128,931 |
| Pneumothorax | 17,313 | 2663 | 167,665 |
| Pleural effusion | 75,696 | 9419 | 102,526 |
| Pleural other | 2441 | 1771 | 183,429 |
| Fracture | 7270 | 484 | 179,887 |
| Support devices | 105,831 | 898 | 80,912 |
