# Supplementary material for: Assessment of the Robustness of Convolutional Neural Networks in Labeling Noise by Using Chest X-Ray Images From Multiple Centers
Source: JMIR Med Inform. 2020 Aug 4;8(8):e18089. doi: 10.2196/18089 (PMC7435602; doi:10.2196/18089)
Supplement: Multimedia Appendix 4 [file medinform_v8i8e18089_app4.docx]

**Multimedia Appendix 4.** Distribution of 3 randomly shuffled datasets.

Normal to normal: Normal labels that have not been changed after random shuffling.

Normal to abnormal: Normal labels that have been changed as abnormal.

Abnormal to normal: Abnormal labels that have been changed as normal.

Abnormal to abnormal: Abnormal labels that have not been changed after random shuffling.

| AMC^a^ and SNUBH^b^ | | 0% | | 1% | | 2% | | 4% | | 8% | | 16% | | 32% | |
| --- | --- | --- | --- | --- | --- | --- | --- | --- | --- | --- | --- | --- | --- | --- | --- |
| Normal to normal | Abnormal to normal | 4974 | 0 | 4912 | 49 | 4586 | 109 | 4729 | 203 | 4522 | 435 | 4141 | 941 | 3448 | 2022 |
| Normal to abnormal | Abnormal to abnormal | 0 | 6112 | 62 | 6063 | 118 | 6003 | 245 | 5909 | 452 | 5677 | 833 | 5171 | 1526 | 4090 |
| NIH^c^ | | 0% | | 1% | | 2% | | 4% | | 8% | | 16% | | 32% | |
| Normal to normal | Abnormal to normal | 5018 | 0 | 4974 | 67 | 4923 | 127 | 4816 | 241 | 4612 | 481 | 4191 | 947 | 3375 | 1905 |
| Normal to abnormal | Abnormal to abnormal | 0 | 6068 | 44 | 6001 | 202 | 5827 | 202 | 5827 | 406 | 5587 | 827 | 5121 | 1643 | 4163 |
| CheXpert | | 0% | | 1% | | 2% | | 4% | | 8% | | 16% | | 32% | |
| Normal to normal | Abnormal to normal | 6112 | 0 | 6042 | 41 | 5994 | 104 | 5891 | 222 | 5631 | 406 | 5132 | 794 | 4127 | 1563 |
| Normal to abnormal | Abnormal to abnormal | 0 | 4974 | 70 | 4933 | 118 | 4870 | 221 | 4752 | 481 | 4568 | 980 | 4180 | 1985 | 3411 |

^a^AMC: Asan Medical Center.

^b^SNUBH: Seoul National University Bundang Hospital.

^c^NIH: National Institutes of Health.
