# Supplementary material for: Assessment of the Robustness of Convolutional Neural Networks in Labeling Noise by Using Chest X-Ray Images From Multiple Centers
Source: JMIR Med Inform. 2020 Aug 4;8(8):e18089. doi: 10.2196/18089 (PMC7435602; doi:10.2196/18089)
Supplement: Multimedia Appendix 5 [file medinform_v8i8e18089_app5.docx]

**Multimedia Appendix 5.** Dataset description for training, validation, and test sets of the Asan Medical Center (AMC) and Seoul National University Bundang Hospital (SNUBH) dataset.

One subject may have multiple abnormalities in given chest x-ray.

|  | Training | | Validation | | Test | |
| --- | --- | --- | --- | --- | --- | --- |
| Hospital | AMC^a^ | SNUBH^b^ | AMC | SNUBH | AMC | SNUBH |
| Normal | 4239 | 735 | 615 | 100 | 1213 | 200 |
| ND^c^ | 710 | 1129 | 102 | 138 | 200 | 249 |
| CS^d^ | 424 | 844 | 84 | 93 | 145 | 177 |
| IO^e^ | 230 | 892 | 32 | 119 | 50 | 211 |
| PLE^f^ | 1144 | 940 | 169 | 100 | 286 | 262 |
| PT^g^ | 282 | 755 | 57 | 112 | 82 | 216 |
| Not Normal | 2564 | 3548 | 403 | 490 | 721 | 955 |
| Total subjects | 6803 | 4283 | 1018 | 590 | 1934 | 1155 |

^a^AMC: Asan Medical Center.

^b^SNUBH: Seoul National University Bundang Hospital.

^c^ND = nodule.

^d^CS = consolidation.

^e^IO = interstitial opacity.

^f^PLE = pleural effusion.

^g^PT = pneumothorax.
