# Supplementary figures and images for: Assessment of the Robustness of Convolutional Neural Networks in Labeling Noise by Using Chest X-Ray Images From Multiple Centers
Source: JMIR Med Inform. 2020 Aug 4;8(8):e18089. doi: 10.2196/18089 (PMC7435602; doi:10.2196/18089)

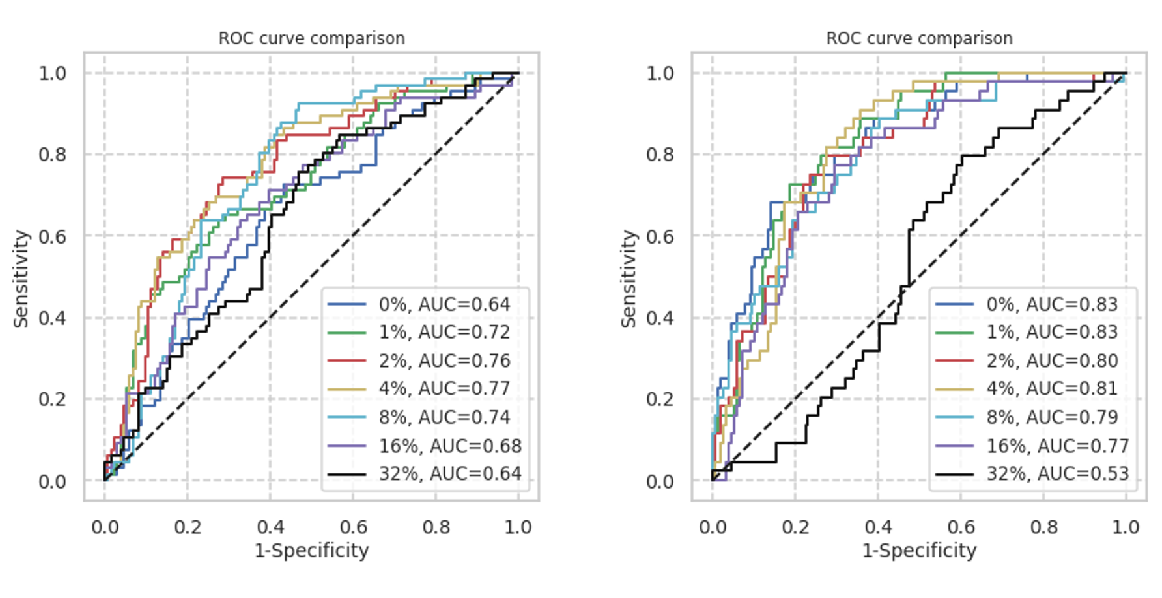

Supplement: Multimedia Appendix 6 [file medinform_v8i8e18089_app6.png]
